# Mammographic density defined by higher than conventional brightness threshold better predicts breast cancer risk for full-field digital mammograms

**DOI:** 10.1186/s13058-015-0654-4

**Published:** 2015-11-18

**Authors:** Tuong Linh Nguyen, Ye Kyaw Aung, Christopher Francis Evans, Choi Yoon-Ho, Mark Anthony Jenkins, Joohon Sung, John Llewelyn Hopper, Yun-Mi Song

**Affiliations:** Melbourne School of Population and Global Health, Centre for Epidemiology and Biostatistics, University of Melbourne, Level 3, 207 Bouverie Street, Carlton, VIC 3053 Australia; Center for Health Promotion, Samsung Medical Center, Sungkyunkwan University School of Medicine, 25-2 Sungkyunkwan-ro, Jongno-gu, 110-745 Seoul, Korea; Seoul Department of Epidemiology and Institute of Health and Environment, School of Public Health, Seoul National University, 1 Gwanak-ro, Gwanak-gu, 151-742 Seoul, Korea; Department of Family Medicine, Samsung Medical Center, Sungkyunkwan University School of Medicine, 25-2 Sungkyunkwan-ro, Jongno-gu, 110-745 Seoul, Korea

## Abstract

**Introduction:**

When measured using the computer-assisted method CUMULUS, mammographic density adjusted for age and body mass index predicts breast cancer risk. We asked if new mammographic density measures defined by higher brightness thresholds gave better risk predictions.

**Methods:**

The Korean Breast Cancer Study included 213 women diagnosed with invasive breast cancer and 630 controls matched for age at full-field digital mammogram and menopausal status. Mammographic density was measured using CUMULUS at the conventional threshold (*Cumulus*), and in effect at two increasingly higher thresholds, which we call *Altocumulus* and *Cirrocumulus*, respectively. All measures were Box-Cox transformed and adjusted for age, body mass index, menopausal status and machine. We used conditional logistic regression to estimate the change in Odds PER standard deviation of transformed and Adjusted density measures (OPERA). The area under the receiver operating characteristic curve (AUC) was estimated.

**Results:**

Corresponding *Altocumulus* and *Cirrocumulus* density measures were correlated with *Cumulus* measures (r approximately 0.8 and 0.6, respectively). *Altocumulus* and *Cirrocumulus* measures were on average 25 % and 80 % less, respectively, than the *Cumulus* measure. For dense area, the OPERA was 1.18 (95 % confidence interval: 1.01−1.39, *P* = 0.03) for *Cumulus*; 1.36 (1.15−1.62, *P* < 0.001) for *Altocumulus*; and 1.23 (1.04−1.45, *P* = 0.01) for *Cirrocumulus*. After fitting the *Altocumulus* measure, the *Cumulus* measure was no longer associated with risk. After fitting the *Cumulus* measure, the *Altocumulus* measure was still associated with risk (*P* = 0.001). The AUCs for dense area was 0.59 for the *Altocumulus* measure, greater than 0.55 and 0.57 for the *Cumulus* and *Cirrocumulus* measures, respectively (*P* = 0.001). Similar results were found for percentage dense area measures.

**Conclusions:**

*Altocumulus* measures perform better than *Cumulus* measures in predicting breast cancer risk, and *Cumulus* measures are confounded by *Altocumulus* measures. The mammographically bright regions might be more aetiologically important for breast cancer, with implications for biological, molecular, genetic and epidemiological research and clinical translation.

## Introduction

Historically, the incidence and prevalence of breast cancer has been lower in Asian countries than Western countries [1, 2]. However, this is changing rapidly with economic development over the past few decades and is expected to increase over the next 20 years [3, 4]. Identification of predictors of risk for Asian women could be an important tool in breast cancer control, especially if they can be readily measured.

Mammographic density is one of strongest risk factors for breast cancer [5, 6]. Conventionally, it has been defined by the white or bright, as distinct from dark, areas on a mammogram. A well-established measurement uses the computer-assisted thresholding method CUMULUS, in which the observer visually selects a pixel threshold to define the dense areas for each particular mammogram [7–9].

In establishing the evidence for mammographic density as a predictor of disease [5, 10], considerable and warranted attention has been made to having observers ‘see’ density in a similar and repeatable way. New observers have been trained to ensure comparability and repeatability with previous observers to measure what has conventionally been referred to as the ‘mammographically dense’ regions of the breast.

Multiple studies of Western women, and a few of Asian women, have shown that, after adjusting for age and body mass index (BMI), the standard measure of mammographic density above predicts breast cancer risk [11–16]. It is important to adjust for age and BMI because these mammographic density measures decrease with increasing age, and with increasing BMI, yet breast cancer risk increases with these factors [17, 18].

We used a Korean case-control study to assess if using in effect a higher than conventional pixel threshold to define density better discriminates cases from controls, i.e. better predicts risk of breast cancer. We assessed the relative discrimination by fitting the density measures based on different degrees of brightness both independently, and together.

We also represented the strength of association for each measure by a new approach, Odds PER Adjusted standard deviation (OPERA), which considers risk gradients for measured variables as a function not of the standard deviation of the *unadjusted* risk factor, as has been conventional practice, but of the standard deviation of that factor after adjusting for all other factors taken into consideration, either by design or analysis, in the case-control comparison [6]. The reason for this is that the correct interpretation of a risk estimate is the change per unit of that factor holding all other factors constant. Therefore it is obvious that the risk per *unadjusted* standard deviation is not the appropriate scale, which should be based on the distribution of that risk factor once it has been adjusted for all relevant covariates.

## Methods

### Subjects

As previously described [19], cases and controls were selected from women who underwent a periodic health checkup at the Health Promotion Center in the Samsung Medical Center, Korea, between February 2006 and December 2011. Breast cancer cases were selected based on a medical record review after breast cancer screening with a mammogram. For each breast cancer case, we chose approximately three controls matched for age (within 1 year), menopausal status, and date of health examination (within 1 month) randomly selected from women who had undergone the same routine health checkup. All controls had no evidence of malignant disease for at least 1 year after the routine health checkup. This study involved 213 breast cancer cases and 630 matched controls. The median age at mammography was 51.5 years and 45 % were under the age of 50 years. This study was approved by the Institutional Review Board of Samsung Medical Center (2011-0013545 and 2014R1A2A2A01002705) [19]. All women gave written consent [19].

### Mammographic density measurements

Mammographic images were obtained using the processed full-field digital mammography system (Senograph 2000D/DMR/DS, General Electric Company, Milwaukee, WI, USA or Selenia, Hologic Inc., Marlborough, MA, USA) in the same institution. We used the cranio-caudal (CC) view of the breast, and for cases, the breast contralateral to that involved in the cancer diagnosis. All measures were conducted in sets of 100, plus a 10 % random repeat sample from within the set (to estimate the intra-class correlation within a set), and in every fifth set, plus the 10 % random sample from the first set (to estimate the intra-class correlation between sets). All measurements were blinded to case-control status as in [19] and blinded to the previous measures.

Mammographic density was measured first using the conventional approach for defining dense areas, and we call those measures *Cumulus*, and they were conducted by TLN, YKA, and CEF. The black or dark areas are not included. TLN’s measures were used in our previous publication [19].

**Fig. 1:**
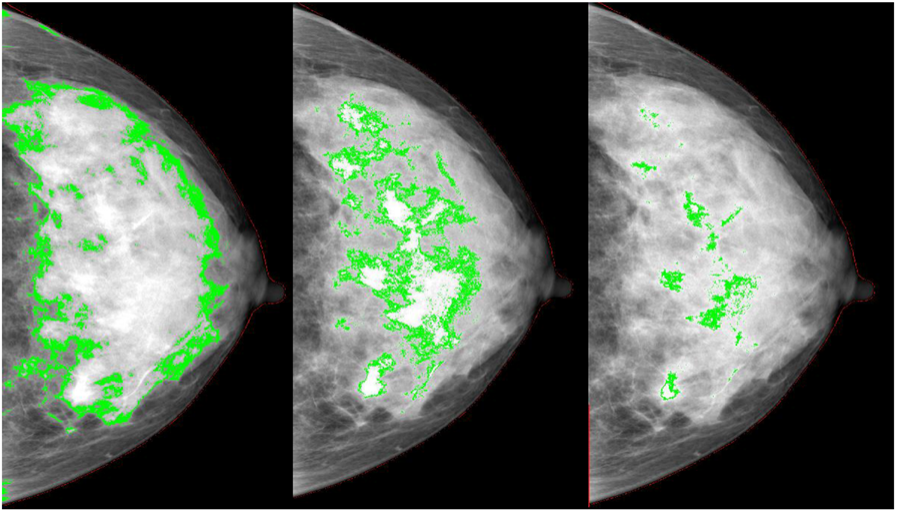
Example of *Cumulus* (*left*), *Altocumulus* (*middle*) *Cirrocumulus* (*right*) measurements from the same image using the CUMULUS software package. For *Cumulus, Altocumulus* and *Cirrocumulus* measures, respectively, the dense area (percentage dense area) was: 716,702 pixels (58 %); 268,374 pixels (22 %); and 51,475 pixels (4 %)

Two of the same observers, TLN and YKA, re-measured all mammograms. This time the observers chose the bright, as distinct from white, areas to be ‘dense’ and therefore in effect defined mammographic density at a higher threshold. The grayish areas that are usually selected when measuring *Cumulus* were not included. We call these latter measures *Altocumulus*. TLN then measured all mammograms using in effect an even higher level of pixel intensity based on what were considered to be only the brightest regions. We call this measure *Cirrocumulus*. The intra-class correlation coefficients for the *Altocumulus* and *Cirrocumulus* measures of dense area were 0.93 and 0.80 cf. 0.98 for the *Cumulus* measure [19]. Figure 1 shows an example of *Cumulus*, *Altocumulus* and *Cirrocumulus* measures from the same mammogram.

### Other measurements

Height and weight, and hence BMI, as well as family history of breast cancer among first-degree relatives, menstrual and reproductive history, and health-related behaviours were measured as described previously [19].

### Statistical analysis

We used the Box-Cox power function to transform the mammographic density measures so that their residuals after adjusting for age and menopausal status (due to the design), BMI (due to negative confounding), and machine (due to technological differences) were approximately normally distributed. We found that the cube root transformation was appropriate for the *Cumulus* and *Altocumulus* dense area measures and a logarithmic transformation was appropriate for the *Cirrocumulus* measure. A cube root transformation was appropriate for their respective percentage dense areas.

We evaluated the association between mammographic density and breast cancer risk by fitting conditional logistic regression models, adjusting for machine (due to different sampling ratios of cases and controls), with the mammographic density measures as both continuous and categorical variables. For the latter, we categorized the transformed mammographic measures, adjusted for age, menopausal status, BMI and machine, into four levels based on the quartile distribution of subjects in the control group, so as to be consistent with the OPERA concept; see below.

We estimated the mammographic density risk associations as the change in log odds per standard deviation of the age, menopausal status, BMI and machine adjusted measures so as to produce values of OPERA [6, 20]. Therefore the risk estimates refer to change in odds per standard deviation of mammographic density adjusted for age, BMI, menopausal status and machine, not of cross-sectional unadjusted mammographic density as is conventionally done, so we derived the former measures, standardized them, and fitted them in our models. Let *r* be the correlation between two mammographic density measures, Y_1_ and Y_2_. Since the standard deviation of Y_j_ adjusted for Y_k_ is SD_j||k_ = [(1 – *r*^2^)]^0.5^, j,k = 1,2, when Y_j_ is fitted with Y_k_ we multiplied the log(OR) estimate from fitting Y_j_ by SD_j|k_ and then exponentiated to obtain the appropriate OPERA (see Appendix).

Statistical analyses, including generation of the receiver operating curves and estimates of and tests of the differences between areas under the receiver operating curves (AUCs) using the DeLong test, and use of the likelihood ratio criterion to test the relative goodness-of-fit of nested models, were performed using the STATA software package [21]. Nominal statistical significance was, by convention, taken to be *P* = 0.05.

## Results

Table 1 shows that the mean age at breast cancer diagnosis for the cases was 51.6 years and 45 % were diagnosed before the age of 50 years, and that 63 % of cases and controls were premenopausal.

**Table 1:** Characteristics of the case and control samples

	Cases (*n* = 213) mean (SD)	Controls (*n* = 630) mean (SD)	*P* ^a^
Age at mammogram (years)	51.6 (7.6)	51.5 (7.4)	0.9
Body mass index (kg/m^2^)	22.5 (2.7)	22.6 (2.8)	0.8
Age at menarche (years)	14.6 (1.6)	14.6 (1.6)	0.9
Number of live birth (per child)	2.11 (0.75)	2.27 (0.91)	0.02
Menopausal status (n, %)			0.9
Premenopausal	134 (62.9)	395 (62.7)	
Postmenopausal	79 (37.1)	235 (37.3)	
Benign breast lump (n, %)			<0.0001
Yes	34 (16.0)	36 (5.6)	
No	179 (84.0)	594 (94.4)	
Ever smoking (n, %)			0.08
Yes	17 (8.0)	30 (4.8)	
Never	196 (92.0)	600 (95.2)	
Ever alcohol consumption (n, %)			0.04
Yes	95 (44.6)	232 (36.8)	
Never	118 (55.4)	398 (63.2)	
Physical exercise (n, %)			0.2
More and equal 90 mins per week	85 (39.9)	223 (35.4)	
Less than 90 mins per week	128 (60.1)	407 (64.6)	
Ever use of hormonal therapy (n, %)			0.3
Yes	33 (15.5)	82 (13.0)	
Never	180 (84.5)	548 (87.0)	
Mammographic measurements			
*Cumulus*			
Dense area (cm^2^)	18.1 (14.9)	15.6 (11.7)	0.01
Non-dense area (cm^2^)	84.3 (36.0)	85.3 (34.2)	0.7
Percentage dense area	18.6 (12.3)	16.2 (10.3)	0.006
Total area (cm^2^)	102.3 (37.3)	100.9 (35.0)	0.6
Density thresholds (0 to 4095)	2174 (355)	2142 (343)	0.2
*Altocumulus*			
Dense area (cm^2^)	14.3 (11.6)	11.4 (8.4)	0.0002
Non-dense area (cm^2^)	85.0 (35.3)	87.5 (34.7)	0.4
Percentage dense area	15.3 (10.8)	12.4 (8.5)	0.0001
Total area (cm^2^)	99.2 (36.2)	99.0 (34.8)	0.9
Density thresholds (0 to 4095)	2247 (301)	2252 (300)	0.8
*Cirrocumulus*			
Dense area (cm^2^)	3.5 (3.4)	3.0 (2.3)	0.03
Non-dense area (cm^2^)	98.1 (41.0)	97.7 (38.8)	0.9
Percentage dense area	3.8 (2.8)	3.3 (2.4)	0.03
Total area (cm^2^)	101.6 (41.4)	100.8 (39.0)	0.8
Density thresholds (0 to 4095)	2559 (205)	2574 (226)	0.4

*SD* standard deviation

^a^ *P* refers to statistical significance for the discrimination between cases and controls

For both cases and controls, the *Altocumulus* measures for dense and percentage dense area were 20–25 % less than the corresponding *Cumulus* measures (all *P* < 0.001); see Table 1. For dense area (percentage dense area), the differences were 4.1 cm^2^ (3.7 %) between *Cumulus* and *Altocumulus* measures, and 13.1 cm^2^ (13.4 %) between *Cumulus* and *Cirrocumulus* measures, respectively. The correlations were 0.84 and 0.79 for *Cumulus* and *Altocumulus*, 0.63 and 0.56 for *Cumulus* and *Cirrocumulus*, and 0.59 and 0.54 for *Altocumulus* and *Cirrocumulus*, respectively.

Table 1 shows that, for *Cumulus, Altocumulus* and *Cirrocumulus*, the mean of the dense and percentage dense areas differed between cases and controls (all *P* < 0.05). The statistical significance was greater for the *Altocumulus* measures (all *P* < 0.001).

Table 2 shows there were significant risk gradients for dense and percentage dense areas after adjusting for covariates (all *P* < 0.05). The OPERA estimates and the AUCs were highest for *Altocumulus*: 1.36 (95 % confidence interval (CI): 1.15–1.62, *P* < 0.001) for dense area and 1.41 (1.19–1.68, *P* < 0.001) for percentage dense area, respectively. The corresponding OPERA estimates for *Cumulus* were 1.18 (1.01–1.39, *P* = 0.03) for dense area and 1.23 (1.05– 1.44, *P* = 0.01) for percentage dense area, respectively. For *Cirrocumulus* they were 1.23 (1.04–1.45, *P* = 0.01) for dense area and 1.21 (1.03–1.42, *P* = 0.02) for percentage dense area, respectively.

**Table 2:** Breast cancer risk association (OPERA) for the mammographic measurements after adjusted for age, body mass index, menopausal status and machine (Hologic and General Electric)

	Cases	OR^a^	95 % CI^b^	*P* ^c^	AUC^d^ (95 % CI)	*LL*
*Cumulus*						
Dense area						
Q1^e^ (*n* = 211)	47	1.00	−	−		
Q2^e^ (*n* = 211)	54	1.18	0.76−1.84	0.5		
Q3^e^ (*n* = 211)	53	1.17	0.74−1.84	0.5		
Q4^e^ (*n* = 210)	59	1.37	0.88−2.14	0.2		
OPERA	213	1.18	1.01−1.39	0.03	0.55 (0.51−0.59)	-290.1865
Percent density						
Q1	49	1.00	−	−		
Q2	49	0.98	0.63−1.54	0.9		
Q3	53	1.09	0.70−1.71	0.7		
Q4	62	1.4	0.90−2.17	0.1		
OPERA	213	1.23	1.05−1.44	0.01	0.56 (0.52−0.61)	-289.0511
*Altocumulus*						
Dense area						
Q1	43	1.00	−	−		
Q2	55	1.46	0.91−2.34	0.1		
Q3	52	1.38	0.85−2.22	0.2		
Q4	63	1.84	1.13−2.99	0.01		
OPERA		1.36	1.15−1.62	<0.001	0.59 (0.55−0.63)	-285.9140
Percentage density						
Q1	42	1.00	−	−		
Q2	54	1.49	0.92−2.39	0.1		
Q3	44	1.16	0.70−1.92	0.6		
Q4	73	2.49	1.52−4.09	<0.001		
OPERA	213	1.41	1.19−1.68	<0.001	0.60 (0.56−0.65)	-284.7104
*Cirrocumulus*						
Dense area						
Q1	41	1.00	−	−		
Q2	58	1.61	1.01−2.58	0.05		
Q3	53	1.42	0.89−2.26	0.1		
Q4	61	1.78	1.11−2.86	0.02		
OPERA	213	1.23	1.04−1.45	0.01	0.57 (0.52−0.61)	-289.3243
Percentage density						
Q1	49	1.00	−	−		
Q2	51	1.06	0.67−1.67	0.8		
Q3	52	1.09	0.70−1.69	0.7		
Q4	61	1.40	0.89−2.22	0.1		
OPERA	213	1.21	1.03−1.42	0.02	0.56 (0.52−0.61)	-289.6855

*OPERA* Odds PER Adjusted standard deviation, *LL* log likelihood

^a^Odds ratio per standard deviation of the risk factors adjusted for age, body mass index (BMI), menopausal status and machine (Hologic and General Electric)

^b^CI = confidence interval

^c^ *P* refers to statistical significance of the odds ratio (OR) estimate

^d^AUCs refer to the area under the receiver operating characteristic curves for mammographic measurements after adjusted for age, body mass index, menopausal status and machine (Hologic and General Electric)

^e^Quartiles (Q1-Q4) defined by distribution of the measure adjusted for age, body mass index, menopausal status and machine (Hologic and General Electric)

Table 3 shows the results from fitting the corresponding *Altocumulus, Cirrocumulus* and *Cumulus* measures together. From the OPERA estimates and standard errors, and from examining the change in log likelihood (*LL*) and AUCs, it was apparent that after fitting the *Altocumulus* measure the addition of the *Cumulus* or *Altocumulus* measures did not improve the fit (*P* > 0.05). On the other hand, from Tables 2 and 3 it can be seen that addition of the *Altocumulus* measure gave a better fit than the *Cumulus* or *Altocumulus* measures alone (*P* = 0.001).

**Table 3:** Estimates of OPERA, 95 % confidence intervals (95 % CI) from fitting multiple mammographic density measures together, correlation between estimates (*R*) and log likelihood (*LL*) for dense area and percentage dense area

	OPERA (95 % CI)	*P*	*R*	*LL*
Dense area				
*Cumulus* ^a^	0.73 (0.53−1.00)	0.05	-0.87	-280.34
*Altocumulus* ^a^	1.83 (1.30−2.57)	0.001		
*Cumulus* ^b^	1.07 (0.88−1.30)	0.5	-0.6	-285.22
*Cirrocumulus* ^b^	1.19 (0.97−1.47)	0.1		
*Altocumulus* ^c^	1.32 (1.07−1.65)	0.01	-0.62	-282.17
*Cirrocumulus* ^c^	1.04 (0.85−1.29)	0.7		
*Cumulus* ^d^	0.71 (0.52−0.98)	0.04	-0.79	-279.97
*Altocumulus* ^d^	1.77 (1.24−2.51)	0.002	-0.19	
*Cirrocumulus* ^d^	1.10 (0.89−1.36)	0.4	-0.22	
Percentage dense area				
*Cumulus* ^a^	0.87 (0.66−1.14)	0.3	-0.81	-280.79
*Altocumulus* ^a^	1.59 (1.18−2.14)	0.002		
*Cumulus* ^b^	1.15 (0.96−1.38)	0.1	-0.50	-284.74
*Cirrocumulus* ^b^	1.14 (0.94−1.37)	0.2		
*Altocumulus* ^c^	1.39 (1.13−1.70)	0.002	-0.51	-284.65
*Cirrocumulus* ^c^	1.03 (0.86−1.25)	0.7		
*Cumulus* ^d^	0.87 (0.66−1.15)	0.3	-0.74	-284.165
*Altocumulus* ^d^	1.55 (1.14−2.10)	0.005	-0.15	
*Cirrocumulus* ^d^	1.05 (0.87−1.27)	0.6	-0.23	

*OPERA* Odds PER Adjusted standard deviation

^a^ *Cumulus* and *Altocumulus* measures fitted together

^b^ *Cumulus* and *Cirrocumulus* measures fitted together

^c^ *Altocumulus* and *Cirrocumulus* measures fitted together

^d^ *Cumulus*, *Altocumulus* and *Cirrocumulus* measures fitted together

**Fig. 2:**
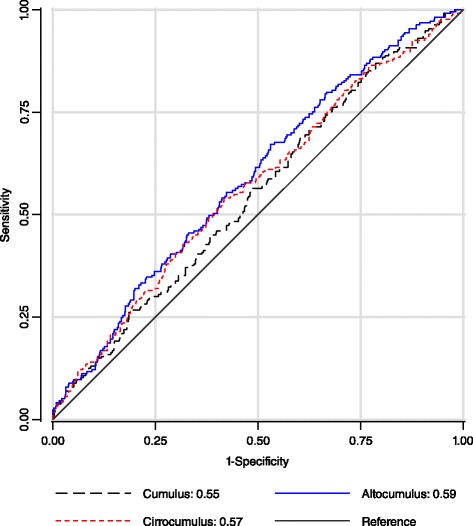
Receiver operating characteristic curve plot of sensitivity against 1-specificty for breast cancer risk, and area under the curve (AUC), for the various dense area measures from full-field digital mammograms in terms of breast cancer risk: Health Promotion Center in the Samsung Medical Center, Korea

Figure 2 shows that, for dense area, the AUCs were: 0.55 (95 % CI 0.51–0.59); 0.59 (0.55–0.63); 0.57 (0.52–0.61) for the *Cumulus*, *Altocumulus* and *Cirrocumulus* measures, respectively. The AUCs for the *Altocumulus* measures were highly significantly greater than for the corresponding *Cumulus* measures (*P* = 0.001). For dense area, the change in AUC from 0.55 for the *Cumulus* measure to 0.59 for the *Altocumulus* measure is 80 % when compared with the baseline AUC of 0.5 corresponding to no association, and this is reflected in a similar change in the log (OPERA) estimates. Similar AUCs applied to the percentage dense area measurements.

## Discussion

We have introduced two new measures of mammographic density, *Altocumulus* and *Cirrocumulus* (Nguyen et al: Mammographic density defined by higher than conventional pixel brightness thresholds better predicts breast cancer risk, submitted), based on defining the mammographically ‘dense’ regions by being successively brighter, and therefore in effect at higher pixel brightness thresholds, than has been convention, which we call *Cumulus*. All density measures discriminated between cases and controls. The risk gradients and AUCs for the dense area and percentage dense area measurements were the same as well as their AUC.

Even though these density measures were correlated, the *Altocumulus* measure performed better than the *Cumulus* and *Cirrocumulus* measures. Moreover, when measures were fitted together, the risk gradient for the *Altocumulus* measure remained statistically significant, while the risk gradient for the *Cumulus* and *Cirrocumulus* measures were reduced and no longer statistically significant. This implies that the apparent risk relationship from traditional *Cumulus* measures has been confounded by the true causes being in breast tissue seen at higher thresholds of pixel intensity, so that the white but not bright areas on a mammogram do not appear to be associated with risk.

Therefore, measuring density at a higher pixel threshold appears to capture more risk-predicting information than measuring at the usual threshold. This is important for several reasons. First, in terms of clinical relevance, we studied digital images, so our findings are relevant to mammography as it is now and will be conducted across most of the world. Digital mammography makes possible automated measures that can be used to provide information in real time. While different measures of ‘breast density’ from digital mammograms are being developed and applied (e.g. [22]), at the moment their only clinical use is to identify women most prone to have a breast cancer missed due to ‘masking’, rather than those at increased risk of a future breast cancer. Our findings inform future developments of automated measures. They also highlight that the two issues – masking and risk prediction – need to be considered separately. While the *Altocumulus* measures of dense area appear to be better predictors of risk, the *Cumulus* measures of percent dense area might be better predictors of masking.

Second, these findings suggest that the mammographically denser regions might be more aetiologically important for breast cancer. The relevant tissues and biological processes involved in explaining why ‘mammographic density’ is a risk factor for breast cancer are more likely to be in the higher density areas of the breast. If confirmed, this is a critical observation for molecular, genetic and other studies trying to determine the underlying biological processes behind this phenomenon [23]. It is also important for research and translation on the prospect of using ‘mammographic density’ to better predict women for interventions or targeted screening.

Third, *Altocumulus* is one of the strongest yet known risk factors for breast cancer when viewed on a population, as distinct from individual, perspective. OPERA is an omnibus measure for discrimination between cases and controls similar to the area under the receiver operator curve, but has the advantage of explicitly taking into account other risk factors. The OPERA we estimated here of 1.4–1.5 for *Altocumulus* is comparable to that for a risk score based on the current common genetic markers (SNPs) recently found to be associated with risk [24]. In comparison, the OPERA for rare mutations in *BRCA1* and *BRCA2*, combined, is only about 1.2, while the OPERA for number of live births is close to 1.1 in a Western population [6].

Obviously there must be an optimal threshold, at least for a given population measured on a given machine by the same observer. This study suggests that it is at a higher pixel level than has been convention, at least for digital mammograms and Korean women. While we are not claiming that *Altocumulus*, as we have measured it, is necessarily the optimal measure, we have shown that the current threshold is not optimal. More research is needed to clarify the situation, especially if automated measures can be developed that allow for changing the threshold. We are currently measuring mammographic density across different thresholds in different populations, and using multiple observers, to try to obtain better mammographic predictors of risk. We encourage others to try varying thresholds to help clarify this important issue.

We are also measuring the familial aggregation of *Altocumulus* and *Cirrocumulus* using twin and family studies, we have done for *Cumulus* [17, 25, 26]. We aim to study the associations of genetic variants known to be associated with breast cancer risk with the *Altocumulus* and *Cirrocumulus* measures, and compare these findings to those for *Cumulus* measures (e.g. [27]).

There are several limitations to this study. The *Cumulus*, *Altocumulus* and *Cirrocumulus* measures depend on the observers. However, given that measurements are performed blind to case-control status, the main issue is repeatability, and all were highly repeatable, the most for *Cumulus* measures. Also, the concepts of “bright” and brightest” regions is somewhat subjective, and can vary across observers. But we have tried to see if and how risk prediction depends on the *threshold*, so the key issue was to have *measurements* in effect at different thresholds (and of course conducted blind to case-control status) and then use OPERA, log likelihoods and AUC to assess the relative goodness of fits.

## Conclusions

This case-control study found that better discrimination between women with and without breast cancer can be achieved by defining mammographic density at a higher pixel brightness threshold than conventional, at least for Asian women. A new measure, *Altocumulus*, performed better than the conventional measure, *Cumulus*, in predicting breast cancer risk from digital mammography images. This suggests that the mammographically denser (bright) regions might be more aetiologically important for breast cancer, with implications for biological, molecular, genetic and epidemiological research and clinical translation. More research is required to work out which threshold is optimal and we encourage other researchers to work on this question.

## Appendix

For each transformed density measure, Y, we fitted a regression for the mean as a linear function of age and BMI and the other fitted covariates. For individual i, E[Y_i_] = b_0_ + b_age_.age_i_ + b_BMI_. BMI_i_ + b_1_X_1i_ + … + b_n_X_ni_, where Y_i_ is their observed value; E[.] represents the expected value; and age_i_, BMI_i_, X_1i_, …, X_ni_ are their age, BMI_i_, and other fitted covariates; and b_0_, …, b_n_ are the corresponding regression coefficients. We then divided the residuals, R_i_ = Y_i_ – E[Y_i_], by the standard deviation of the residuals, SD(R_i_), to give Y’ = Ri/SD(Ri).

The different density measures, Y’, were fitted independently and then together; the improvement in fit was assessed using the likelihood ratio test. When we fitted two density measures, Y1’ and Y2’, into the same model, we presented the risk estimates in terms of the change in the standard deviation after adjusting also for the other measure, to be consistent with the OPERA concept. Let *r* be the correlation between Y_1_’ and Y_2_’. Because the standard deviation of Y_j_^’^ adjusted for Y_k_’ is SD’ = [(1 – *r*^2^)]^0.5^, for j,k = 1,2, when Y_j_’ is fitted with Y_k_’ we multiplied the log(OR) estimate from fitting Y_j_’ by SD’ and then exponentiated it to obtain the appropriate OPERA. [For *r* = 0.6, 0.7, and 0.8, SD’ approximately 0.8, 0.7, and 0.6, respectively].

## Abbreviations

AUC: area under the receiver operating curve
BMI: body mass index
CI: confidence interval
*LL*: log likelihood
OPERA: Odds PER Adjusted standard deviation
*P*: refers to statistical significance of the odds ratio estimate
*R*: correlation between estimates
*r*: correlation
SD_j|k_: standard deviation of Y_j_ adjusted for Y_k_
